# Optimizing outcomes of partial nephrectomy in patients with tumors in solitary kidneys: a non-systematic review

**DOI:** 10.25122/jml-2025-0066

**Published:** 2025-04

**Authors:** Stelian Ianiotescu, Constantin Gingu, Alexandru Iordache, Adrian Preda, Osama Salloum, Irina Balescu, Nicolae Bacalbasa, Ioanel Sinescu

**Affiliations:** 1 Carol Davila University of Medicine and Pharmacy, Bucharest, Romania; 2 Center of Uronephrology and Kidney Transplantation, Fundeni Clinical Institute, Bucharest, Romania; 3 Department of Nephrology, Urology, Immunology and Immunology of Transplant, Dermatology, Allergology, Faculty of Medicine, Carol Davila University of Medicine and Pharmacy, Bucharest, Romania; 4 Department of Surgery, Carol Davila University of Medicine and Pharmacy, Bucharest, Romania; 5 Department of Visceral Surgery, Center of Excellence in Translational Medicine, Fundeni Clinical Institute, Bucharest, Romania; 6 Department of Visceral Surgery, Center of Digestive Diseases and Liver Transplantation, Fundeni Clinical Institute, Bucharest, Romania

**Keywords:** Solitary kidney, renal cell carcinoma, nephron-sparing surgery, thermal ablation, radiofrequency ablation, stereotactic ablative body radiotherapy, immune checkpoint inhibitors, artificial intelligence, preoperative planning, postoperative outcome prediction

## Abstract

This review consolidates recent evidence on managing renal cell carcinoma (RCC) in patients with solitary kidneys. It provides a comprehensive discussion of evolving strategies in partial nephrectomy—including open, laparoscopic, and particularly robot-assisted partial nephrectomy (RAPN)—along with thermal and advanced ablative therapies, non-surgical options (such as stereotactic ablative body radiotherapy [SABR] and active surveillance [AS]), and emerging neoadjuvant systemic treatments with tyrosine kinase inhibitors (TKIs) and immune checkpoint inhibitors (ICIs). In addition, the integration of artificial intelligence (AI) for preoperative planning, intraoperative guidance, and postoperative outcome prediction is discussed. Given the limited renal reserve in these patients, preserving functional renal parenchyma is paramount. This multidisciplinary review synthesizes evidence from 2018 to the present and is supported by 70 contemporary references.

## INTRODUCTION

Renal cell carcinoma (RCC) accounts for approximately 2–3% of adult malignancies worldwide [1,2]. With the increased use of advanced imaging techniques such as multidetector computed tomography (MDCT) and magnetic resonance imaging (MRI), there has been a notable rise in the incidental detection of renal masses, particularly small lesions [3-17]. In patients with a solitary functioning kidney (SFK)—whether due to congenital absence, previous contralateral nephrectomy, or severe parenchymal atrophy—the management of RCC poses unique challenges. Even minimal loss of renal parenchyma in these high-risk patients may precipitate rapid deterioration in kidney function, substantially increasing the likelihood of chronic kidney disease (CKD) and progression to end-stage renal disease (ESRD) that may necessitate dialysis or transplantation [3,4,18]. Partial nephrectomy (PN), also referred to as nephron-sparing surgery (NSS), is the treatment of choice for localized RCC (typically clinical stage T1, ≤7 cm) in patients with SFK because it strives to achieve complete oncologic resection while preserving as much healthy renal tissue as possible [5]. Over recent decades, the surgical practice has evolved from conventional open partial nephrectomy (OPN) to minimally invasive techniques, with robot-assisted partial nephrectomy (RAPN) emerging as a leading modality due to its enhanced three-dimensional visualization, improved instrument dexterity, and superior ergonomics [6,7]. In parallel, thermal ablation methods (including cryoablation, radiofrequency ablation [RFA], and microwave ablation [MWA]) and non-surgical modalities like SABR and AS have broadened the therapeutic arsenal. Additionally, neoadjuvant systemic treatments with TKIs and ICIs are under investigation to downstage tumors preoperatively, and the growing incorporation of artificial intelligence (AI) into imaging interpretation, surgical navigation, and outcome prediction holds promise for further individualizing therapy [8–15].

## MATERIAL AND METHODS

The literature research was conducted between 1 January 2025 and 20 March 2025, utilizing the PubMed database to explore advancements in the management of renal cell carcinoma (RCC) in patients with a solitary kidney. The study focused on interventions such as partial nephrectomy (including open and robot-assisted approaches), thermal ablation techniques (radiofrequency ablation, cryoablation), stereotactic ablative body radiotherapy, active surveillance, and neoadjuvant therapy with immune checkpoint inhibitors. Artificial intelligence applications in treatment planning or outcomes were also investigated.

The keywords used for the research were renal cell carcinoma, solitary kidney, partial nephrectomy, nephron-sparing surgery, open partial nephrectomy, robot-assisted partial nephrectomy, thermal ablation, radiofrequency ablation, cryoablation, microwave ablation, stereotactic ablative body radiotherapy, active surveillance, neoadjuvant therapy, immune checkpoint inhibitors, and artificial intelligence.

Studies published from 1 January 2018 to 10 March 2025 were reviewed, with inclusion criteria limited to all English-language study types. Articles irrelevant to the research objective or not in English were excluded. Initial screening identified 881 articles, which were narrowed down to 120 during the first selection. Following further evaluation of relevance and quality, 70 articles were ultimately selected for inclusion. These articles were analyzed to synthesize current evidence, technological advancements, and clinical recommendations for managing RCC in solitary kidney patients, emphasizing the balance between oncological efficacy and renal functional preservation.

### Epidemiology of renal tumors in solitary kidneys

Renal cell carcinoma ranks among the top 15 most common malignancies globally [18], with a steadily increasing incidence largely attributed to the widespread use of advanced cross-sectional imaging modalities, such as MDCT and MRI, which facilitate the incidental detection of renal masses [16,17]. In patients with a solitary functioning kidney (SFK)—whether due to congenital absence, prior contralateral nephrectomy, or severe parenchymal atrophy— the clinical stakes are considerably higher. Even a minor loss of nephron mass in these patients can trigger a rapid decline in renal function, significantly increasing the risk of progressing to chronic kidney disease (CKD) and end-stage renal disease (ESRD), which may ultimately necessitate dialysis or transplantation [3,4,18]. Histologically, clear cell RCC (ccRCC) is the predominant subtype, while papillary and chromophobe variants are less common. Key modifiable risk factors include cigarette smoking, obesity, and hypertension. In addition, genetic mutations—particularly in the *VHL, MET*, and *SETD2* genes—have been implicated in RCC pathogenesis (Table 1) [19-21]. Population-based studies underscore that even slight reductions in renal tissue in SFK patients have profound long-term clinical and socioeconomic implications, emphasizing the urgent need for therapeutic strategies that preserve as much renal parenchyma as possible [22–25].

**Table 1:** Summary of main genetic alterations in renal cell carcinoma

RCC subtype	Chromosomal abnormalities	Gene mutations / Molecular alterations
Clear Cell RCC	Loss of 3p (including the *VHL* gene) Loss of the Y chromosome Gains on 5q, 8q, 4p, 14q, and 9p	Inactivation/mutations of *VHL* Mutations in *PBRM1, SETD2, BAP1*, and *KDM5C*
Papillary RCC (Type I)	Trisomy of chromosome 7 (including 7q31)	Overexpression/activating mutations of *MET*; additional alterations may involve other genes (e.g., *VHL, PBRM1, SETD2, BAP1, KDM5C*)
Papillary RCC (Type II)	Loss of the Y chromosome Gains on 8q; losses on 1p and 9p	More aggressive molecular profile, including silencing of *CDKN2A* and additional epigenetic changes
Chromophobe RCC	Losses of chromosomes 1, 2, 6, 7, 10, 13, 17 (more common in classical subtype) Gains on chromosomes 4, 7, 11, 12, 14q, and 18q	Frequent mutations in *PTEN, TP53, mTOR, c-kit* Other alterations (e.g., *FAAH2, PDHB, PDXD1, ZNF765, PRKAG2, ARID1A, ABHD3*)

### Surgical management: resection techniques and outcomes

Surgical resection remains the cornerstone of curative treatment for localized RCC in SFK patients, with the critical challenge being complete oncologic resection combined with maximum renal parenchyma preservation. Traditionally, open partial nephrectomy (OPN) has been the standard approach, especially for centrally located or complex tumors. Shahzad *et al*. [1] reported that central tumors often necessitate complex dissection and rigorous vascular control, resulting in higher postoperative morbidity. Thompson *et al*. [4] further quantified that each additional minute of warm ischemia during OPN leads to a measurable loss of renal function. Although OPN is associated with greater blood loss, longer hospital stays, and prolonged recovery [26-28], it remains indispensable for cases requiring extensive resection. The advent of laparoscopic partial nephrectomy (LPN) marked the initial move towards minimally invasive surgery, reducing morbidity compared to OPN; however, the technical challenges of LPN—such as intracorporeal suturing—limit its utility in highly complex cases [29-31]. Robot-assisted partial nephrectomy (RAPN) has revolutionized nephron-sparing surgery by offering enhanced three-dimensional visualization, improved instrument dexterity, and better ergonomics [32,33]. These advantages enable more precise tumor excision while facilitating advanced ischemia-sparing techniques, such as selective clamping and off-clamp resections, which reduce warm ischemia time and help preserve renal function [34-38]. Intraoperative modalities, including ultrasound and near-infrared fluorescence imaging, further improve tumor localization and margin assessment [32,33,38-41]. Moreover, validated clinical decision-making tools, such as the RENAL and PADUA nephrometry scores [6], support individualized preoperative planning and risk stratification, facilitating the selection the most appropriate surgical approach for each patient with an SFK [39-44]. Advancements in three-dimensional virtual modeling and augmented reality (AR) overlays have also enhanced surgical simulation and planning, helping to optimize resection strategies in this high-risk population [12,13,42].

### Ablative therapies and non-surgical modalities

For patients with small renal masses or those deemed high risk for surgical intervention, ablative therapies, and non-surgical modalities offer important treatment alternatives that emphasize the preservation of renal parenchyma. Thermal ablation techniques such as cryoablation and radiofrequency ablation (RFA) have been thoroughly evaluated. Studies conducted so far demonstrate that these modalities yield oncologic outcomes comparable to partial nephrectomy while preserving renal function [17,43]. However, precise post-ablation imaging is essential for assessing the ablated volume and predicting long-term renal outcomes [28,29]. For patients unsuitable for invasive procedures, non-surgical options like stereotactic ablative body radiotherapy (SABR) and active surveillance (AS) have shown substantial promise. SABR delivers high-dose, conformal radiation in a few sessions, achieving durable local control with minimal toxicity [30,42]. Similarly, active surveillance—supported by percutaneous renal mass biopsy and quantitative imaging—allows for careful monitoring and timely intervention for small, indolent tumors, thereby avoiding overtreatment [19-21]. Neoadjuvant therapy further expands treatment options; tyrosine kinase inhibitors (TKIs) have been shown to reduce tumor dimensions by 10–28% or more, increasing the feasibility of partial nephrectomy from 65% in the pre-TKI era to 90–91% after neoadjuvant TKI therapy, with meta-analyses reporting a relative risk of 1.84 for successful nephron-sparing surgery [44-52]. Immune checkpoint inhibitors (ICIs) similarly induce significant histopathologic changes that enhance surgical resectability even when radiologic shrinkage is modest [6,11]. Combination regimens of ICIs with TKIs (IO/TKI) have demonstrated promising synergistic effects. One case report noted that the integration of neoadjuvant combination therapy rendered a previously unresectable tumor resectable, thereby preserving renal function and preventing dialysis [7,10]. Despite these advances, further prospective randomized trials are necessary to standardize neoadjuvant regimens and validate predictive biomarkers [1,4,7,11,51,52].

### Integration of artificial intelligence in management

AI is increasingly integrated into RCC management for SFK patients, enhancing preoperative planning, intraoperative guidance, and postoperative outcome prediction. AI-driven algorithms can automatically segment renal masses, vasculature, and the collecting system from CT and MRI scans, creating detailed three-dimensional virtual models that facilitate surgical simulation and planning [12,13]. During surgery, augmented reality systems overlay these virtual models onto live video feeds, providing real-time visualization of tumor margins and critical anatomical structures, which enables more precise resection and selective clamping [42,52-69]. Moreover, machine learning models that analyze comprehensive preoperative, intraoperative, and postoperative data have demonstrated potential in predicting complications such as acute kidney injury (AKI) and long-term declines in estimated glomerular filtration rate (eGFR), enabling personalized postoperative management strategies [40,41]. Future advances are expected to leverage multimodal data—including genomics and clinical records—to refine outcome predictions and enhance treatment individualization [69,70].

### Comparative outcome analysis and prognostic considerations

The overriding objective in managing RCC in SFK patients is to achieve effective oncologic control while preserving renal function. Techniques that minimize warm ischemia, such as selective clamping during RAPN, result in smaller declines in eGFR, with reported reductions of 3–5% following RAPN compared to 7–8% (or more) following OPN [14,35,63,64,65]. Ablative modalities that avoid vascular clamping entirely generally preserve near-baseline renal function, although these techniques are best suited for small lesions and require close post-procedural imaging surveillance [66]. Oncologic outcomes, including recurrence-free survival (RFS) and cancer-specific survival (CSS), are broadly comparable among partial nephrectomy, RAPN, and ablative therapies. Some studies suggest that RAPN may be associated with slightly lower positive surgical margin rates due to enhanced precision [8,10]. Conversely, certain ablative techniques may confer a modestly increased risk of local recurrence for tumors larger than 3 cm, underscoring the importance of meticulous patient selection and rigorous follow-up imaging [47-49,53,54]. Additionally, minimally invasive approaches like RAPN are consistently associated with reduced intraoperative blood loss, decreased transfusion requirements, and shorter hospital stays, all contributing to improved recovery and overall quality of life—an especially critical consideration for SFK patients [2,3,10,45,70]. Integrating validated preoperative decision aids and advanced imaging further ensures that treatment is individualized to achieve the optimal balance between oncologic efficacy and renal preservation [6,28,29].

### Neoadjuvant systemic therapies

Neoadjuvant systemic therapies are emerging as an important strategy to downstage tumors preoperatively, thus enhancing the feasibility of nephron-sparing surgery in SFK patients with complex RCC. Tyrosine kinase inhibitors (TKIs) targeting vascular endothelial growth factor receptor (VEGFR)—including agents like sunitinib, pazopanib, and axitinib—have demonstrated tumor downsizing effects ranging from 10% to 28% or more, simplifying the surgical field and reducing warm ischemia time. Studies indicate that the introduction of neoadjuvant TKI therapy has raised the successful rate of partial nephrectomy from 65% in the pre-TKI era to approximately 90–91%, with meta-analyses yielding a relative risk of 1.84 for successful nephron-sparing surgery [4,5]. Neoadjuvant immune checkpoint inhibitors, such as nivolumab, although resulting in modest radiographic tumor size reductions, induce significant histopathologic changes—including enhanced tumor necrosis and inflammatory infiltration—that improve surgical resectability [6,11]. Furthermore, combination regimens of ICIs with TKIs (IO/TKI) show promising synergistic effects that may further downstage aggressive tumors, making nephron-sparing surgery more feasible [7,10]. Nonetheless, variability in perioperative outcomes exists, and further prospective studies are needed to refine treatment protocols and identify predictive biomarkers [1,4,7,11,52,51]. However, “the future of neoadjuvant therapy in RCC hinges upon rigorous clinical validation and the integration of novel biomarkers into clinical decision-making.”

## DISCUSSION

The management of RCC in patients with solitary kidneys is uniquely challenging, as preserving the limited renal parenchyma is vital for preventing CKD and ESRD. Surgical resection approaches have evolved from traditional OPN to minimally invasive techniques such as robot-assisted partial nephrectomy (RAPN), significantly improving visualization, instrument dexterity, and ischemia-sparing strategies. Data show that minimizing warm ischemia time is critical for renal function preservation, and modern techniques such as selective clamping have enabled RAPN to achieve impressive outcomes even in complex cases [32-37]. Importantly, studies indicate that even when warm ischemia extends beyond 33 minutes, preservation of renal parenchyma is a more decisive factor in determining long-term renal function than the ischemia duration alone [4,14,35].

Ablative therapies such as cryoablation and RFA have demonstrated oncologic control rates comparable to PN for small renal masses while preserving renal function by avoiding vascular clamping. Nevertheless, a modestly increased risk of local recurrence for tumors larger than 3 cm has been reported, highlighting the importance of strict patient selection and meticulous imaging follow-up [47-49,53,54]. For patients at high surgical risk, non-surgical modalities such as SABR and active surveillance are viable alternatives that provide high local control rates with minimal toxicity and preserve renal function [56-62].

Neoadjuvant systemic therapies present an exciting frontier by enabling tumor downstaging prior to surgery. TKIs have been shown to reduce tumor size significantly, thereby increasing the feasibility of nephron-sparing surgery from 65% in the pre-TKI era to 90–91% in recent cohorts [4,5]. Similarly, immune checkpoint inhibitors induce histopathologic changes that enhance resectability, and combination regimens (IO/TKI) have exhibited synergistic benefits [7,10]. However, further prospective research is needed to standardize these protocols and incorporate robust predictive biomarkers into clinical practice [1,4,7,11,51,52].

Integrating AI into RCC management—from preoperative planning using 3D imaging to intraoperative augmented reality guidance and postoperative predictive analytics—is revolutionizing the field. AI-enhanced systems facilitate precise identification of tumor margins and critical anatomical structures, enhancing surgical accuracy and reducing complication rates. These advanced technologies and multidisciplinary collaboration are essential to optimizing individualized treatment strategies that balance oncologic control with renal preservation (Table 2) [12,13,42,69,70].

**Table 2:** Comparison of treatment modalities for RCC in solitary kidneys

Characteristic	Open Partial Nephrectomy (OPN)	Robot-Assisted Partial Nephrectomy (RAPN)	Ablative Therapies (Cryo/RFA/MWA)	Stereotactic Ablative Radiotherapy (SABR)	Active Surveillance (AS)	Neoadjuvant Therapy
Mechanism/Approach	Open surgical resection	Minimally invasive, robotic-assisted resection	In situ thermal destruction (percutaneous/laparoscopic)	Targeted high-dose radiation delivered in a few sessions	Non-invasive imaging-based monitoring; biopsy if needed	Systemic administration of drugs (TKIs/ICIs) preoperatively to shrink tumor and downstage disease
Tumor suitability	Complex, centrally located tumors	T1 tumors (≤7 cm) with variable complexity	Small tumors (<3–4 cm); preferably peripheral	Small tumors; patients deemed non-surgical	Small tumors (<3 cm), slow growth; selected patients	Locally advanced or borderline resectable tumors; cases where downstaging may increase the feasibility of PN
Invasiveness/Morbidity	High (significant blood loss, longer hospital stay, prolonged recovery)	Moderate (minimally invasive with reduced morbidity)	Minimal (percutaneous/laparoscopic approach)	Minimal (percutaneous/laparoscopic approach)	Non-invasive	Non-invasive (biopsy possible)
Impact on ischemia	Frequent global warm ischemia (variable warm ischemia time)	Minimal ischemia (selective/off-clamp techniques; reduced warm ischemia time)	No ischemia	No ischemia	Not applicable	Improves feasibility of PN by reducing tumor size, thereby indirectly preserving renal parenchyma
Renal function preservation	Moderate to good (dependent on warm ischemia time and residual parenchyma)	Very good (due to reduced ischemia and high precision)	Excellent (no clamping required)	Excellent (highly precise targeting)	Maximal preservation (no surgical intervention)	Improves feasibility of PN by reducing tumor size, thereby indirectly preserving renal parenchyma
Oncologic control	Excellent	Excellent (potential for improved negative margins)	Good to excellent (risk increases with tumors >3 cm)	Good to excellent (local control achieved)	Acceptable risk of progression	Potentially improves resectability and long-term oncologic outcomes when combined with surgery; variable responses
Key advantages	Standard approach for handling extreme complexity	Minimally invasive, high precision, reduced ischemia, rapid recovery	Minimally invasive; no ischemia; repeatable	Non-invasive; avoids general anesthesia	Avoids treatment-related morbidity	May render unresectable tumors resectable; reduces tumor size and warm ischemia time; may treat micrometastatic disease
Key limitations	High morbidity, slower recovery, and greater blood loss	Requires specialized expertise and technology; higher cost	Limited efficacy for larger tumors; requires careful follow-up	Limited long-term data; no pathological confirmation; cost considerations	Risk of tumor progression/metastasis; potential patient anxiety	Systemic toxicities; variable response; requires careful patient selection and standardization of protocols
Follow-up requirements	Standard imaging protocols	Standard imaging protocols	Frequent and meticulous imaging	Regular imaging assessments to evaluate treatment response	Regular imaging and clinical monitoring	Regular imaging to assess tumor response and routine monitoring of systemic therapy side effects

## CONCLUSION

The integration of advanced surgical (minimally invasive, nephron-sparing) and non-surgical (ablative, systemic) therapies with AI-driven planning and imaging enhances oncologic and functional outcomes for patients with solitary kidney tumors, reducing CKD risks. Ongoing research remains crucial to standardize protocols and validate long-term benefits.
